# Dr. D. Braden Kyle

**Published:** 1916-11

**Authors:** 


**Dr. D. Braden Kyle,** Eclectic of Cincinnati, 1891, died at his home in Philadelphia, Oct. 23, aged 63, of pneumonia. He was widely known as a laryngologist and as an author.

